# Microbiome shifts with onset and progression of Sea Star Wasting Disease revealed through time course sampling

**DOI:** 10.1038/s41598-018-34697-w

**Published:** 2018-11-07

**Authors:** Melanie M. Lloyd, Melissa H. Pespeni

**Affiliations:** University of Vermont, Department of Biology, 109 Carrigan Drive, Burlington, Vermont 05405 USA

## Abstract

The recent outbreak of Sea Star Wasting Disease (SSWD) is one of the largest marine epizootics in history, but the host-associated microbial community changes specific to disease progression have not been characterized. Here, we sampled the microbiomes of ochre sea stars, *Pisaster ochraceus*, through time as animals stayed healthy or became sick and died with SSWD. We found community-wide differences in the microbiomes of sick and healthy sea stars, changes in microbial community composition through disease progression, and a decrease in species richness of the microbiome in late stages of SSWD. Known beneficial taxa (*Pseudoalteromonas* spp.) decreased in abundance at symptom onset and through disease progression, while known pathogenic (*Tenacibaculum* spp.) and putatively opportunistic bacteria (*Polaribacter* spp. and *Phaeobacter* spp.) increased in abundance in early and late disease stages. Functional profiling revealed microbes more abundant in healthy animals performed functions that inhibit growth of other microbes, including pathogen detection, biosynthesis of secondary metabolites, and degradation of xenobiotics. Changes in microbial composition with disease onset and progression suggest that a microbial imbalance of the host could lead to SSWD or be a consequence of infection by another pathogen. This work highlights the importance of the microbiome in SSWD and also suggests that a healthy microbiome may help confer resistance to SSWD.

## Introduction

Traditionally, symptom progression of infectious diseases has been thought to be the result of one pathogenic organism infecting a host. Researchers are now starting to understand the complexities of the microbial ecology within hosts through disease symptom progression. Some diseases are now known to be the result of infection by a suite of pathogenic microbes (i.e., polymicrobial diseases^1^) or a general disruption of the host’s microbiome (i.e., dysbiosis^2^). For example, Black Band Disease of corals, a model polymicrobial disease, is the result of infection by a community of microbes which all play a role in disease progression^3^. Polymicrobial diseases of humans are also being recognized with increasing frequency and researchers are finding that symptom progression of some diseases of known etiology, such as measles^4^ and pneumonia^5^, are more complicated than previously considered. Some chronic human diseases may be the result of dysbiosis, including inflammatory bowel disease, multiple sclerosis, type I diabetes, allergies, and asthma^2^. Understanding the role of all interacting microbial players that lead to disease progression is becoming increasingly important as large scale epidemics increase in frequency in humans^6^ and other species, particularly in marine populations^7,8^. Here we investigate the role of the microbiome in a keystone species, the sea star *Pisaster ochraceus*^9^, through disease progression of Sea Star Wasting Disease (also called Sea Star Wasting Syndrome or Asteroid Idiopathic Wasting Syndrome), one of the largest marine epizootics in recorded history^10,11^.

Sea Star Wasting Disease refers to a suite of morphological signs of disease affecting more than 20 species in the class Asteroidea, including the ochre sea star, *Pisaster ochraceus*^11^. Relatively small-scale SSWD events have been observed on the west coast of North America since the 1980s^12–14^. Beginning in 2013, SSWD was observed at an unprecedented scale in terms of the geographic range and number of taxa affected as well as extended duration of the event^11,15–17^. The geographic extent of the recent SSWD event (2013-present) is from Prince William Sound, Alaska to Baja California, Mexico, completely encompassing the geographic range of many affected species^11^ (see citizen science database of all recorded SSWD observations: http://data.piscoweb.org/marine1/seastardisease.html). Morphological signs of SSWD include loss of turgor pressure, deflating and twisting rays, lesions, disintegrating tissue, ray autotomy, and often death of the affected individual^11^. For *P*. ochraceus, the iconic keystone predator^9^, population declines have been recorded along nearly the entire species range. SSWD is still ongoing throughout the range and population recovery remains uncertain, particularly in southern regions^18^. SSWD is a significant threat to some of the most ecologically important predatory species (i.e. *P. ochraceus* and *Pycnopodia helianthoides*) in the intertidal and subtidal zones along the west coast of North America. Their removal from both of these ecosystems has had a dramatic, direct effects on their prey populations with likely long-lasting indirect effects on inter- and subtidal communities^16,19–23^.

The etiology of SSWD remains unresolved^10^. A group of single-stranded DNA viruses, the wasting asteroid-associated densoviruses (WAaDs), is associated with SSWD signs in one species, *P*. *helianthoides*, but not others^10^. For *P. ochraceus*, challenges with virus size fractionated material (i.e., <0.2 µm) from SSWD-affected individuals did not elicit disease signs^10^ and the health status of individuals (asymptomatic or symptomatic) showed no relationship to the abundance of WAaDs^10,11^. Additionally, other factors often linked to the onset of marine diseases, such as water temperature and population density, have not been linked to SSWD in the 2013-present outbreak^10,18^. Sites vary in the relationship between temperature and SSWD: in Oregon, SSWD increased with cooler temperatures^16^, whereas in Washington, SSWD increased with warmer temperatures^15^. Thus, it seems plausible that SSWD is not the result of a single etiology across the entire geographic and taxonomic ranges, but rather the result of a combination of environmental factors, unidentified pathogens, changes in the host’s immune system, and/or changes in host-associated bacterial community.

Little work has been done to understand the bacterial taxa involved in SSWD (but see Gudenkaug and Hewson^24^). Given the importance of microbes in health and disease, the main objective of this study was to understand the potential role of the sea star microbiome in the onset and progression of SSWD. We used repeated time-course sampling of initially asymptomatic adult *P. ochraceus* maintained in individual aquaria as they naturally progressed through SSWD and compared their microbial community composition to samples from stars collected from the same population and maintained in the same conditions that remained healthy. This experimental design controlled for the variation in microbiota between individuals to allow us to test several hypotheses: (1) that there are microbial community differences between sick and healthy sea stars, (2) that there is a remodeling and eventual simplification of the bacterial community through disease progression, and (3) that time course sampling of healthy and sick animals can reveal beneficial, putatively pathogenic, and opportunistic microbes in disease.

## Results

### Disease progression and microbiome characterization

**Figure 1:**
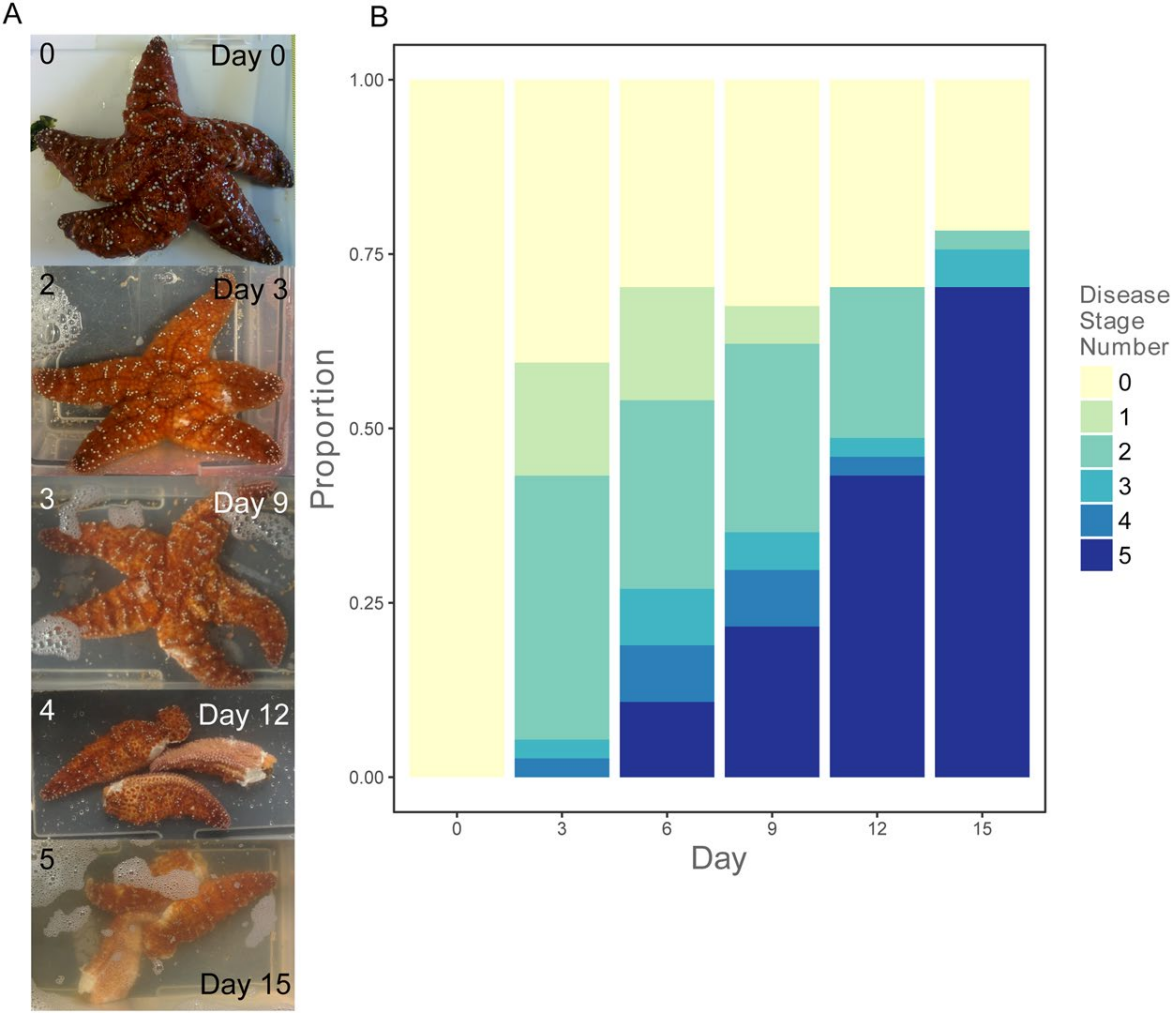
Sea Star Wasting Disease progression through the two-week experiment. (**A**) Photographs taken from one *P. ochraceus* individual as it progressed through SSWD. Numbers in the top left corner of each picture relate to the *P. ochraceus* SSWD symptom guide from seastarwasting.org with the addition of category 5 for dead individuals. (**B**) Proportion of the 37 individuals of each symptom number at the six sampling time points.

For two weeks, thirty-seven field-collected, adult *P. ochraceus* were kept in individual aquaria in a temperature-controlled incubator and monitored for signs and progression of SSWD. All experimental animals were asymptomatic at the time of collection and on arrival to the lab. The sea stars were collected in an area that had previously experienced SSWD, Monterey, CA, and thus contained a suite of microbes representative of a SSWD-exposed population. We chose Monterey, CA because the site had been impacted by SSWD, and because the site is centrally located along the species range. We did not experimentally infect individuals, rather some sea stars presented symptoms during the two-week experiment while others did not. Every three days until death or the end of the experiment, nonlethal tissue samples (3.5 mm diameter biopsy punches of epidermal tissue) were taken from each individual. At each sampling time point, individuals were inspected for SSWD signs which were classified based on severity according to the *P. ochraceus* SSWD symptom guide as follows: (0) healthy; (1) one lesion on one ray or the central body; (2) lesions on two rays, one ray and the central body, or deteriorating rays; (3) lesions on most of the body and/or one or two missing rays; (4) severe tissue deterioration and/or three or more missing rays; (5) dead^25^. Eight individuals remained healthy to the end of the two-week experiment and 29 became sick and eventually died (Fig. 1A,B). RNA was extracted from 176 biopsy tissue samples (83 samples were taken from individuals when they were healthy, 85 samples were taken from individuals when they were sick, and 8 samples were taken from dead individuals, see Supplementary Table 1 for details about each sample). All samples were negative for the SSaDV using the qRT-PCR assay reported by Hewson *et al*.^11^. We used RNA-based amplicon sequencing of the V3-V4 region on the 16S rRNA bacterial gene to characterize microbial community composition of sick and healthy *P. ochraceus*. The 176 libraries were sequenced on an Illumina MiSeq, producing 300 base pair overlapping paired end reads. After quality control and filtering, we identified 1,064 Operational Taxonomic Units (OTUs) represented across all samples based on homology with the Greengenes Database (mean 41,029+/−8,381 reads per library).

### Differences between samples taken from sick and healthy individuals

**Figure 2:**
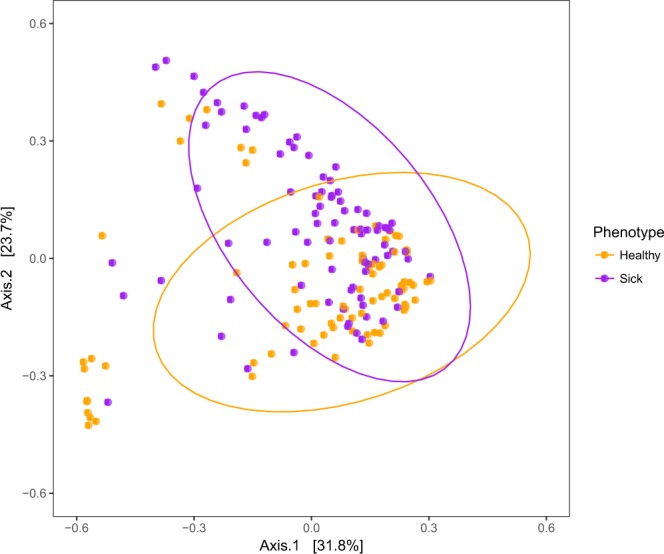
Differences in microbial community between sick and healthy individuals. Principal Coordinate Analysis plots of microbial communities from all samples throughout the experiment, excluding the 8 samples taken from individuals when dead. Samples that were taken from an individual that was sick at the time of sampling are colored purple while samples that were taken from an individual that was healthy at the time of sampling are colored orange. Principal Coordinate Analysis was based on the weighted UniFrac distance matrix. Ellipses are drawn around each group’s centroid (confidence interval, 0.95).

Species richness, the estimated number of microbial taxa (measured as the chao1 alpha diversity index), did not differ between samples taken from individuals that were healthy at the time of sampling versus samples taken from individuals that were sick at the time of sampling (chao1, *t*-statistic = −1.433, *P* = 0.151). However, microbial community composition did differ between these healthy and sick samples using the UniFrac beta diversity metric, which incorporates information on the relative relatedness of community members by incorporating phylogenetic distances between observed taxa^26^ (Fig. 2; permutational multivariate analysis of variance, PERMANOVA, on both unweighted and weighted UniFrac distance matrices, unweighted: pseudo-F statistic = 4.784, *P* = 0.001; weighted: pseudo-F statistic = 15.282, *P* = 0.001). The unweighted UniFrac distance matrix does not take into account differences in taxa abundance and only considers presence/absence while the weighted UniFrac distance matrix does take into account differences in taxa abundance. 208 OTUs (18.3% of the total OTU table) differed in abundance between sick and healthy samples with 95 of the 208 more abundant in samples from healthy versus sick individuals and 113 more abundant in samples from sick versus healthy individuals (Supplementary Table 2; *P*_adj_ < 0.1). OTUs more abundant in healthy samples were most commonly in the *Pseudoalteromonas* genus (16 of 95 OTUs) or were not taxonomically assigned to the level of genus (61 of 95 OTUs). Unassigned OTUs were taxa not represented in the Greengenes Database but were given unique identifiers to track changes in abundance. OTUs more abundant in sick samples were most commonly in the *Tenacibaculum* (10 of 113 OTUs) and *Polaribacter* genera (10 of 113 OTUs) or not assigned to the taxonomic level of genus (57 of 113 OTUs).

To provide insight into the cellular processes associated with the distinct microbial communities of sick versus healthy individuals, we used taxonomy-based functional profiling^27^. We were able to characterize function based on KEGG orthology for 24.2% of the total OTU table (258 of the 1,064 OTUs). We identified 20 pathways enriched in samples taken from healthy individuals and 20 pathways enriched in samples taken from sick individuals (Supplementary Table 3; *P*_adj_ < 0.05). Among these differentially enriched functional classes, the pathway descriptions most commonly enriched for higher abundance in healthy communities were metabolism of secondary metabolites, including flavonoid and neomycin biosynthesis, and degradation of xenobiotics such as atrazine, one of the most commonly used herbicides and a known endocrine disruptor^28^. The pathway descriptions most common among those enriched in sick microbial communities were metabolism of amino acids and energy metabolism (*P*_adj_ < 0.05). Interestingly, communities from both healthy and sick individuals had enriched pathways related to immune function. Microbes from healthy individuals were enriched for pathways related to amoebiasis and RIG-I-like receptor (RLR) signaling. RLRs detect RNA virus infection and elicit innate immune responses^29^. Two pathways related to Vibrio were enriched among the microbes from sick individuals (Supplementary Table 3; *P*_adj_ < 0.05). These results suggest that microbes abundant in healthy animals perform functions that inhibit the growth of other potentially pathogenic microbes.

### Initial differences in microbial community

Comparing samples taken upon arrival, species richness (chao1 alpha diversity) and taxonomic composition (UniFrac beta diversity) did not differ between individuals that ended the experiment healthy vs. those that ended the experiment sick (chao1, t-statistic = −1.112, *P* = 0.26; unweighted PERMANOVA, pseudo-F statistic = 1.467, *P* = 0.114; weighted PERMANOVA, pseudo-F statistic = 1.817, *P* = 0.123). However, despite similarities in overall diversity, 75 OTUs differed in abundance between the initial samples from the 29 individuals that became sick versus the 8 individuals that remained healthy (Supplementary Table 4; *P*_adj_ < 0.1), though this number of differentially abundant OTUs was within the null distribution based on 1000 random permutations. Interestingly, 91% of these taxa (68 OTUs) were in relatively higher abundance in healthy individuals while 9% (7 OTUs) were in higher abundance in sick individuals. Of the 68 OTUs more abundant in healthy animals, 51 were uncharacterized in the Greengenes Database, but match marine taxa previously found in healthy Pacific Oysters, *Crassostrea gigas*, based on BLAST alignment^30^. The 7 OTUs that were higher in abundance in individuals that eventually became sick belonged to the Flammeovirgaceae, Colwelliaceae, or Francisellaceae families, which contain known pathogens^31^.

### Microbial community changes occurring with symptom onset

To identify changes in microbial community that happened as an individual first developed signs of SSWD, we tested for OTUs whose abundance showed an interaction between time and health status in a subset of samples: those taken immediately before and after disease signs were first observed in sick animals paired with corresponding samples in time from healthy animals. This test identified 145 OTUs with changes in abundance (14% of the total OTU table), all of which except one were more abundant in healthy relative to sick animals through time (Supplementary Table 5; *P*_adj_ < 0.1). The most common taxonomic classification of these OTUs was the genus *Pseudoalteromonas* (20 of 145 OTUs).

### Differences through SSWD progression

**Figure 3:**
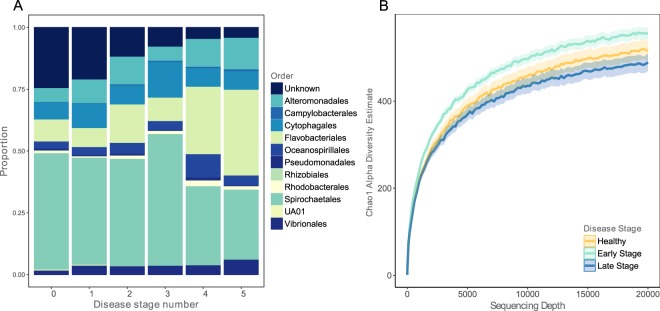
Microbial community differences through SSWD symptom progression. (**A**) Proportions of OTUs classified to the Order level in microbial communities from sea stars sampled through disease stages. Disease stage number relates to the *P. ochraceus* SSWD symptom guide from seastarwasting.org with the addition of a category 5 for dead individuals. (**B**) Rarefaction plot of mean chao1 alpha diversity estimates as a function of sequencing depth for samples grouped by disease stage. Shaded areas represent the standard error of the mean (SEM) for the chao1 estimates.

To identify changes in microbial community that were specific to early and late disease stages, we tested for differences in microbial communities between samples characterized by different disease stage numbers and found that abundance of bacterial orders differed depending on symptom number (Fig. 3A). Through disease progression, there was a decrease in previously uncharacterized bacterial taxa (Fig. 3A, chi-square = 30.78, *P* < 0.001), which could be due to greater research efforts directed towards the characterization of disease-causing rather than healthy microbiota. To increase statistical power to identify specific taxa associated with broader disease stages, symptom numbers 1 and 2 were collapsed to ‘early stage disease’ and numbers 3, 4, and 5 were collapsed into ‘late stage disease’. 83 collected samples were classified as healthy, 69 were classified as early stage disease, and 24 were classified as late stage disease. Beta diversity, i.e., community composition, differed between microbial communities of samples taken from individuals among all disease stages (unweighted PERMANOVA pseudo-F statistic = 2.925, *P* = 0.001; weighted pseudo-F statistic = 8.317, *P* = 0.001). Considering species richness, healthy vs. early stage disease individuals did not differ (chao1, t-statistic = 1.570, *P* = 0.39), nor did healthy vs. late stage disease individuals (chao1, t-statistic = 2.026, *P* = 0.156). However, species richness did differ between early vs. late stage disease individuals where late stage disease individuals had fewer taxa than early stage disease individuals (chao1, *t*-statistic = 4.268, *P* = 0.003, Fig. 3B), suggesting a reduction in the microbial diversity due to an increase in abundance of fewer, likely opportunistic pathogens at late stages of disease.

**Figure 4:**
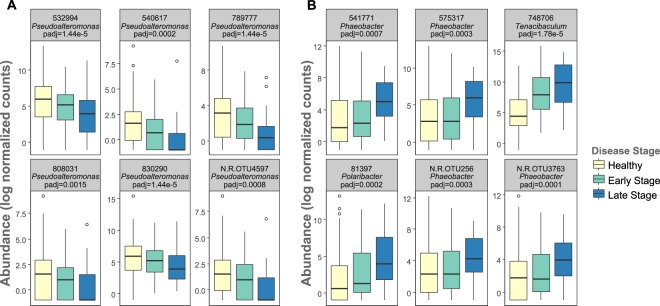
Changes in abundance of OTUs through SSWD progression. (**A**) Log normalized abundance of 6 OTUs whose abundance decreased from healthy samples through symptom progression. Abundance of these OTUs differed between healthy and early stage sick samples (adjusted *P* values presented from this test) and between healthy and late stage sick samples. **(B)** Log normalized abundance of 6 OTUs whose abundance increased from healthy samples through symptom progression. Abundance of these OTUs differed between healthy and early stage sick samples (adjusted *P* value presented from this test) and between healthy and late stage sick samples. The centerline of the boxplots represents the median of the data, the box represents the interquartile range, and the whiskers represent the minimum and maximum values.

**Figure 5:**
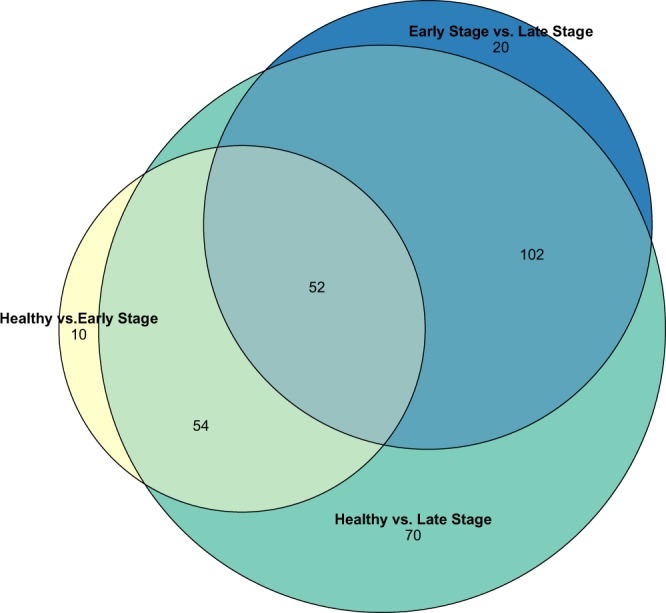
Venn diagram of differentially abundant OTUs between samples of different disease stages. ‘Early-stage disease’ includes symptom numbers 1 and 2 and ‘late-stage disease’ includes symptom numbers 3, 4, and 5.

Healthy and early-stage samples differed in the abundance of 119 OTUs; healthy and late-stage samples differed in the abundance of 278 OTUs; and early- and late-stage samples differed in the abundance of 177 OTUs (Supplementary Tables 6–8, respectively; *P*_adj_ < 0.1). Of the 119 differentially abundant OTUs between healthy and early stage samples, 47 were in higher abundance in healthy samples, 15 of which were *Pseudoalteromonas* spp. (Fig. 4A). Among the 72 OTUs higher in early stage sick samples, the most common genera were *Tenacibaculum* and *Polaribacter* (Fig. 4B). Among the 119 OTUs changing in abundance from healthy to early stage samples, 10 OTU changes were specific to the shift between healthy and early-stage symptoms (Fig. 5). Of these 10 OTUs differentially abundant between healthy and early-stage sick individuals, 8 of them were relatively higher in healthy samples than sick samples and 4 of these were in the *Pseudoalteromonas* genus. Of the 177 differentially abundant OTUs between early stage and late stage sick samples, 138 were in higher abundance in late stage samples (most commonly *Phaeobacter* spp. and *Polaribacter* spp.) and 39 were in higher abundance in early stage samples. Lastly, 20 of the 177 OTUs changing in abundance from early to late stage disease samples were specific to that shift. 12 of these 20 were relatively higher in late stage samples compared to early-stage samples (Fig. 5). The most common genus was *Moritella*, a small genus of marine bacteria, one species of which causes winter ulcer, a disease that primarily affects salmonid fish during cold periods^32^. Many of the taxa (106 OTUs) that differed in abundance between healthy and early stage samples also differed in abundance between healthy and late stage samples (Figs 4 and 5, Supplementary Tables 6 and 7).

## Discussion

The goal of this study was to investigate the potential role of the microbiome in a poorly understood and highly destructive disease. To our knowledge, this is the first characterization of the microbiome during SSW disease progression. Our results suggest that the microbiome plays a role in SSWD in *P. ochraceus*, which is perhaps not surprising given that the microbiome is a key player in many human^1,2^ and nonhuman diseases, including marine diseases^33^, and given the complex biogeography of SSWD spread, the large number of species affected, and the lack of consistent correspondence to the abiotic factors of temperature and precipitation^11^. Results from our time course sampling of the microbiome of healthy and sick sea stars support our hypotheses that there are distinct microbial communities specific to sick and healthy individuals and that there is a remodeling of the microbial community through disease progression, which did not occur in healthy individuals. In addition, the loss of known beneficial microbes and the increase of a suite of known pathogens support the hypothesis that SSWD may be the result of an imbalance in the microbial community, or a dysbiosis disease. This imbalance could result in disease symptoms and or allow for infection by one or multiple pathogens. While cause versus effect is difficult to distinguish without experimental manipulation of microbes, the temporal resolution of this experiment has allowed for the identification of microbial communities specific to healthy animals, early stage disease animals, and late stage disease animals.

An alternative explanation is that these microbial community differences are not causing SSWD symptoms, but are symptoms themselves of another, unidentified pathogen. Recent work has shown that infection rates and mortality driven by single, known pathogens depend on the microbial community composition as shaped by conditions such as temperature and precipitation^34,35^, coinfection with other pathogens^36,37^, or the use of antibiotics, probiotics, fungicides, or pesticides^38,39^. These interactions between pathogen, microbiome, and conditions have been shown in a range of systems, chytridiomycosis in amphibians^34,38^, bovine tuberculosis in African buffalo^36^, and Enterobacterial blooms in gut inflammation diseases in humans and mouse models^37,39^. In sea urchins, which are echinoderms like sea stars, temperature and pH conditions have been shown to impact microbial community composition and affect immune function^40–42^. Other potentially important players in disease are bacteriophages (phages), viruses that infect bacteria, because they can affect microbial community composition and make way for opportunistic pathogens in a host^35,43,44^. They have also been shown to lyse their hosts in temperature-, nutrient-, and density-dependent conditions^35^. This is an area of active research^43^, particularly in the context of coral reef health^44^. Thus, these scenarios, a dysbiosis disease or a single pathogen, may not be mutually exclusive and highlight the interplay between the environment, microbiome, host, and pathogen(s) in disease emergence.

We found a specific signature of microbial community shifts correlating with disease progression in sick stars distinct from the microbial community of healthy stars. This signature was characterized by a decrease in abundance of *Pseudoalteromonas* spp. in sick individuals at the time of first symptom onset (Fig. 3B; Supplementary Table 5), followed by an increase in *Tenacibaculum* spp. and *Polaribacter* spp. bacteria in early stages of disease (Fig. 3C; Supplementary Table 6), followed by an increase in abundance of putatively opportunistic bacteria (*Phaeobacter* spp. and *Polaribacter* spp.) late in disease progression (Supplementary Table 7). Members of the *Pseudoalteromonas* genus live within marine eukaryotes and create a competitive advantage against other microbes by producing anti-bacterial, bacteriolytic, agarolytic, and algicidal molecules^45^. *Pseudoalteromonas* spp. have been considered for use as biological control agents to control pathogens in aquaculture productions of fish, abalone, and oysters^46,47^ and even as a bioactive compound for use in human pharmaceuticals^48^. It is possible that these bacteria are occupying niche space in healthy animals and have a competitive edge against potentially pathogenic microbes, such as *Tenacibaculum* spp. bacteria, a genus containing marine pathogens^49^. In fact, some members of the *Pseudoalteromonas* genus are key members of some coral species’ holobionts and have been shown to be able to regulate the coral bacterial community through antimicrobial activity and kill bacterial taxa suspected of causing coral diseases^50–52^. In a time-course, experimental infection study of white band disease of *Acropora cervicornis* corals, OTUs from the family Alteromonadaceae (which contains the *Pseudoalteromonas* genus) were in abundances that suggested a role as a defensive symbiont of the host coral^53^.

The decrease in abundance of *Pseudoalteromonas* spp. bacteria was followed by the proliferation of *Tenacibaculum* spp. and *Polaribacter* spp., which increased in abundance after first signs of disease but early in progression. These early stage disease changes were then followed by proliferation of putatively opportunistic pathogenic *Phaeobacter* spp. and *Polaribacter* spp. Some *Phaeobacter* species have complex dynamics and switch between a mutualist life strategy and opportunistic pathogen strategy^54^. These results suggest a model for SSWD progression that starts with a decrease in abundance of putatively beneficial bacteria (*Pseudoalteromonas* spp.), followed by an increase in abundance of putatively pathogenic bacteria after symptoms first appear (*Tenacibaculum* spp. and *Polaribacter* spp.), and finally, an increase in abundance of opportunistic pathogens as the disease progresses (*Phaeobacter* spp. and *Polaribacter* spp.; see summary model Supplementary Fig. 1).

We found minimal overlap in the bacterial taxa identified in this study and the previous study of bacterial community members in SSWD-affected *Pycnopodia helianthoides*: of the 20 most abundant bacterial genera identified previously, only 2 (*Pseudomonas* and *Vibrio*) were identified in this study but at much lower frequency^24^. This may be due to the fact that these studies were done in different host species that belong to different families of sea stars. Alternatively, it may be that this most recent SSWD outbreak was not the result of a single disease outbreak or stress event but multiple concordant events^10^. In additions, all of our samples were negative for the Sea Star-associated densovirus (SSaDV) using the diagnostic qRT-PCR assay^11^ despite positive amplification of biological and technical controls. These results add to the growing evidence that SSWD in *P. ochraceus* is not associated with the initially identified SSaDV^10^.

Traditionally, the cause of an infectious disease has been identified by fulfilling Koch’s Postulates: (1) the pathogenic organism is found in diseased individuals and not healthy individuals, (2) the pathogenic organism is isolated in pure culture, (3) the cultured organism causes disease when introduced into a healthy individual, and (4) the organism is again isolated from the inoculated, diseased individual and is identical to the originally isolated pathogen^55–57^. However, this approach might not be appropriate or realistic for polymicrobial diseases^55^. Furthermore, linking marine disease outbreaks to a specific causative pathogen is notoriously difficult^58^. Researchers have suggested modifying Koch’s Postulates in order to accommodate our new and evolving understanding of disease^59^. Sato *et al*.^3^ recommended four steps to understanding pathogenesis of polymicrobial diseases: “…the following factors must be identified: (i) microbial communities that are uniquely and/or commonly present in disease lesions, (ii) key microbial members and their individual functions that contribute to a microenvironment that enables pathogenic communities to develop, (iii) metabolic interactions between the key microbial players that are collectively responsible for pathogenesis, and (iv) ecological factors that alter interactions between the host and its pathogens and predispose the host to formation of the polymicrobial communities”. The results of the present study contribute to fulfilling these steps for SSWD by identifying the members of the microbial communities specific to *P. ochraceus* with and without SSWD. We identified putatively beneficial, pathogenic, and opportunistic bacterial members of the *P. ochraceus* microbial community as they relate to SSWD. Overall, our results suggest that SSWD onset and progression may not be caused by one pathogenic organism but may be the result of a complicated interaction between multiple microbial taxa. To further explore the roles of host genotype and environmental and ecological conditions in shaping host-associated microbial communities, future work should compare microbial communities of healthy and sick stars from adjacent impacted and not impacted sites from multiple species.

Some studies of marine organisms show a shift in microbiome composition as a result of culture in aquaria, on the time scale of days to months to years^60–62^, while other studies show that the microbiome is not affected by transfer to and culture in aquaria^63–65^. In this study, it is possible that handling and maintenance in aquaria had an effect on the microbiomes of these experimental stars. However, such an effect would not change the conclusions of this study due to our experimental design of housing animals in individual aquaria in a common conditions. Eight of the 37 animals remained healthy for the duration of the experiment. If the handling and culture in artificial seawater induced wasting, all individuals would have become sick because all individuals were handled in the same manner. In addition, upon arrival to the lab, there were only small differences in the individuals that became sick vs. those that stayed healthy; samples from these groups were not different in species richness or UniFrac beta diversity and the number of differentially abundant OTUs between these samples were relatively few compared to other contrasts (Supplementary Table 9). Thus, our results suggest that handling alone was unlikely to differentially affect the 37 individuals, causing only some to get sick and die, and suggest that the sea stars came in with the microbial communities and pathogens that ultimately shaped their fate.

In the face of rapidly changing global environmental conditions, epidemics are increasing in frequency and magnitude, and it is important to understand the mechanisms of disease resistance^6,66^. Our results add to the growing body of evidence that supports the hypothesis that the host-associated microbiome could provide a protective benefit to the host or yield hosts more susceptible to infection^67–69^. Our data provide evidence for a community of microbes associated with resistance to SSWD and support the hypothesis that SSWD may be due to a dysbiosis of a healthy microbiome followed by infection by one or multiple pathogenic bacteria. Future studies should test the impacts of environmental pollutants and/or changes in environmental conditions on microbial communities sea star immunity, and signs of wasting. Considering the effects of these keystone predators on the intertidal community, SSWD may be an example of microbial ecology within hosts impacting large-scale community-wide disturbances.

## Methods

### Animal collection and experimental design

Thirty eight adult (mean length from tip of ray to middle of the oral disc, R = 9.7 cm ± 2.2 standard deviation), asymptomatic *P. ochraceus* were collected by hand by SCUBA from around the wharf at Monterey Harbor, Monterey, California (36°36′21.44″N 121°53′23.69″W) on May 4, 2016 or June 8, 2016. *P. ochraceus* had experienced a high incidence of SSWD at Monterey in late 2013 and 2014, but at the time of collection in mid 2016, the majority of individuals were asymptomatic (see http://data.piscoweb.org/marine1/seastardisease.html for a detailed history of SSWD observations in this region). The average temperature at the time of collection was 12 °C. The stars were shipped overnight in individual bags with approximately 300 ml seawater per bag to the University of Vermont; the bags of stars were shipped in a Styrofoam box with a layer of freezer packs on the top and bottom of the box with bunched newspaper between the freezer packs and the stars to buffer against extreme cold. The total transit time was approximately 17 hours for both shipments. Immediately upon arrival, nonlethal biopsy punches were taken from the body wall of each individual (3.5 mm diameter biopsy punch, Robbins Instruments, Chatham, NJ) and individuals were photographed and checked for signs of SSWD. Only one individual showed signs of SSWD upon arrival, individual 30; it was not included in the experiment (Supplementary Table 1). The biopsy sampling method was pilot tested previously and shown not to kill or harm individuals (data not shown). Individuals were transferred to individual, numbered, pre-leached plastic containers (31.5 cm × 18.5 cm × 11.5 cm) filled with 1 gallon of artificial seawater (33 parts per thousand Instant Ocean salt (Blacksburg, VA) mixed with RO water), with individual bubblers, and kept in one of two incubators (SANYO MLR-350, Osaka, Japan) at 12 °C. Every three days, nonlethal biopsy punches were taken from the body wall of each individual. Only epidermal body wall tissue was sampled, even when sampling wasting individuals. For individuals displaying SSW, wasting epidermal tissue, rather than healthy epidermal tissue, was sampled. All biopsy tissue samples were flash frozen in liquid nitrogen in 2 ml tubes and stored at −80 °C. At each sampling time point, photographs were taken and signs of disease were recorded according to the *P. ochraceus* SSWD symptom guide^25^. Every Monday, Wednesday, and Friday containers were manually cleaned and refilled with fresh artificial seawater, then bubbled with the same bubbler. Animals were not fed during the experiment in order to not introduce additional microbes and microbial variation with the food source. The experiment was terminated after fifteen days. Dead individuals were removed from the experiment at the first time they were observed to be dead. If this observation occurred at a sampling time point, a sample was taken and included in the analysis with a phenotype of ‘Dead’ (8 total samples were classified as such).

### RNA extraction and cDNA reverse transcription

RNA was extracted from each biopsy punch using a modified TRIzol protocol (TRIzol reagent ThermoFisher Scientific, Waltham, MA). Tissue was lysed in 250 ul TRIzol with a plastic pestle then homogenized with 750 ul more TRIzol on a Vortex Genie2 (Scientific Industries, Bohemia, NY) for 20 minutes. 200 ul chloroform (ThermoFisher Scientific, Waltham, MA) was added to the mixture which was inverted 15 times, incubated for 3 minutes, and centrifuged for 15 minutes at 12,000 × g at 4 °C. The aqueous phase containing RNA was transferred to a new tube and this step was repeated a second time. The RNA was precipitated from the aqueous phase by the addition of 500 ul isopropanol (ThermoFisher Scientific, Waltham, MA) and 1 ul 5 mg/ml glycogen (Invitrogen, Carlsbad, CA), incubation for 10 minutes at room temperature, and centrifugation for 5 minutes at 7500 × g at 4 °C. The RNA pellet was dried for 10 minutes at room temperature and resuspended in 50 ul nuclease-free water. The quality and quantity of the RNA extractions was measured using a NanoDrop 2000 Spectrophotometer (ThermoFisher Scientific, Waltham, MA) and Qubit 3.0 Fluorometer (Life Technologies, Carlsbad, CA). The RNA was checked for contaminating DNA by negative amplification PCR (see below for 16S PCR amplification parameters). cDNA was reverse transcribed with SuperScript IV (Invitrogen, Carlsbad, CA), using random hexamer primers.

### SSaDV diagnostic qPCR

cDNA from all samples were tested for the presence of SSaDV according to the protocol and primers from Hewson *et al*.^11^. Duplex qRT-PCR reactions tested for the presence of both the nonstructural protein 1 (NS1; forward primer, 5′-ttaaggatcgggttcgtgtc-3′; reverse primer, 5′-tgcaagcggattaggtttct-3′; probe, 5′-tcaattggatgagtgcaccatttttga-3′; oligonucleotide standard, 5′-tttaaggatcgggttcgtgtcttcaattggatgagtgcaccatttttgaagaattatgataagaaacctaatccgcttgcag-3′) and the viral gene product 4 (VP4; forward primer, 5′-ttgcattaattcctgctggt-3′; reverse primer, 5′-tgtaccaccagtgggatagc-3′; probe, 5′-tgatgtcatgcaaactgttgccaca-3′; oligonucleotide standard, 5-tttgcattaattcctgctggtagttacataaagtctgtatctattgatgtcatgcaaactgttgccacaactggctatcccactggtggtacaa-3′). 25 ul reactions were run in duplicate for each sample and contained 80 nM of each primer and probe, 2 ul cDNA, and 1x iTaq Universal Probe Supermix (BioRad, Hercules, CA) (Hewson, I., *personal communication*). The conditions were as follows: 95 °C for 5 minutes followed by 60 cycles of 95 °C for 30 seconds and 55 °C for 30 seconds. qRT-PCR reactions were amplified in a BioRad CFX Connect Real-Time System. Biological and technical positive controls were included. The technical control was a synthetically produced oligonucleotide which contained both oligonucleotide standard sequences reported in Hewson *et al*.^11^. The biological control was a DNA extraction of a SSaDV-positive sea star provided by the Hewson lab.

### 16S PCR amplification and sequencing

The V3 and V4 region of the 16S bacterial gene were amplified with the following primers (Illumina adapter overhang nucleotide sequences are underlined): Forward 5′TCGTCGGCAGCGTCAGATGTGTATAAGAGACAGCCTACGGGNGGCWGCAG and Reverse 5′ GTCTCGTGGGCTCGGAGATGTGTATAAGAGACAGGACTACHVGGGTATCTAATCC^70^. 25 ul PCR reactions (1X MiFi Mix (Bioline, Toronto, Canada), 200 nM each primer, and 2 ul cDNA) were run with the following conditions: 95 °C for 3 minutes followed by 25 cycles of 95 °C for 30 seconds, 55 °C for 30 seconds, and 72 °C for 30 seconds, with a final extension at 72 °C for 5 minutes. PCR products were cleaned with AMPure XP beads (Beckman Coulter, Brea, CA), MiSeq indexing adapters were added to PCR products, and indexed PCR products were cleaned with AMPure XP beads according to the Illumina 16S metagenomic sequencing library preparation protocol^71^. Cleaned, indexed PCR products were run on a 2% agarose gel to check for appropriately sized bands. 16S rRNA gene amplicon libraries were sequenced at RAPID Genomics (Gainesville, Florida, USA) on an Illumina MiSeq platform using 2 × 300 base pair overlapping paired-end reads.

### Sequence data processing and OTU clustering

Sequences were demultiplexed and barcode sequences were removed at RAPID Genomics. Paired end reads were matched and quality filtered using QIIME’s ‘multiple_join_paired_ends.py’ and ‘multiple_split_libraries_fastq.py’, respectively, with default parameters^72^. Sequences were clustered into Operational Taxonomic Units (OTUs) with QIIME’s Open Reference OTU algorithm (a combination of Closed Reference OTU clustering based on 97% similarity to the Greengenes Database followed by *de novo* OTU clustering) using QIIME’s ‘pick_open_reference_otus.py’; default parameters were used except for ‘enable_rev_strand_match,’ which was set to True^72–75^. The clustering step also included taxonomic assignment of *de novo* OTUs using the RDP Classifier to the Greengenes Database version 13_8^76,77^ which matches a centroid sequence of each OTU to the lowest common ancestor (LCA) in the Greengenes Database. The *de novo* assignment commonly results in partial taxonomic classification of *de novo* clustered OTUs. The last step of OTU assignment is assembly of a phylogenetic tree of the taxa in the OTU table with FastTree 2.1.3^78^. The phylogenetic classification of the family Pseudoalteromonadaceae is incorrectly placed in the order Vibrionales in the Greengenes Database^79^. We corrected this assignment to the order Alteromonadales in our phylogenic tree. OTUs present in fewer than 25% of samples were removed using QIIME’s ‘filter_otus_from_otu_table.py’. Chimeric OTUs were filtered using VSEARCH^80^. The above processes resulted in an OTU table containing 1,064 OTUs. To gain additional taxonomic information on some OTUs of interest, a representative sequence of that OTU was used in a BLAST alignment search with default parameters^81^.

### Microbial community analysis

We produced a distance matrix containing a dissimilarity value for each pairwise comparison between samples based on both the weighted and unweighted Unifrac metrics from a rarefied OTU table (rarefied to 20,000 reads/sample) using QIIME’s ‘core_diversity_analyses.py’^26^. We used QIIME’s ‘compare_alpha_diversity.py’ and ‘compare_categories.py’ to test for differences between choa1 alpha diversity and UniFrac beta diversity, respectively. For alpha diversity, we compared results from all three metrics: chao1, observed OTUs, and Faith’s Phylogenetic Diversity, which produced corroborating results. For beta diversity, we compared results from the following methods, which produced corroborating results: adonis, ANOSIM, MRPP, db-RDA, and PERMANOVA each with 999 permutations. To test for community wide differences in microbial communities between different groups of samples, we used the above methods to test for differences in choa1 alpha diversity and UniFrac beta diversity between samples taken from sick vs. healthy individuals, samples taken from healthy individuals vs. early stage disease individuals vs. late stage disease individuals, and samples taken upon arrival comparing individuals which ended the experiment healthy vs. those that ended the experiment sick. Because diversity measures increase with sample size^82^, in comparisons that involved unbalanced sample numbers, we randomly subsampled the group with more samples down to the number of samples in the group with fewer samples. Principal Coordinate Analysis plots based on the Bray-Curtis distance were produced in the R package, Phyloseq^83^.

### Differential expression of OTUs

DESeq2 is commonly used to test for differential expression between genes in RNASeq datasets^84^. This same program can be used to test for differential abundance of OTUs using the Phyloseq package^83,85^. Input to DESeq2 is a matrix of raw counts of each OTU per sample. The counts datum is normalized to account for differences in sequencing depth between libraries. DESeq2 uses a generalized linear model with a negative binomial distribution to test for differential abundance between groups by calculating the Wald’s statistic and *P* value for every OTU in the table. A Benjamin Hochberg multiple test correction accounts for the multiple testing of the many OTUs in the counts table^84^. We tested for differential abundance of OTUs between groups using a number of models as follows. To test for the effect of the health status of the individual at the time of sampling while controlling for the repeated measures of each individual, we used the model ~individual + phenotype and limited the OTU table to either sick or healthy samples (168 samples from N = 37 individuals). To test for differences between individuals that remained healthy vs. individuals that became sick at Day 0, we limited the OTU table to only samples on that day (37 samples from N = 37 individuals) and used the model ~Final_phenotype. To identify differences in OTU abundance that happened as an individual first developed signs of SSWD, we limited the OTU table to samples immediately before and after the first observation of disease in sick individuals as well as corresponding sample pairs from healthy individuals (46 paired before/after samples from N = 23 individuals). Samples from individuals that became sick between the first two sampling points (Day 0 and Day 3) were excluded from this group. We then tested the model ~Final_phenotype + Time + Final_phenotype:Time vs. Final_phenotype + Time. In testing for differential abundance of OTUs between samples taken from individuals of different disease stages, we needed to increase statistical power by collapsing the Pacific Rocky Intertidal Monitoring Program’s disease classification as follows: stage numbers 1 and 2 were collapsed to ‘early disease stage’ and numbers 3, 4, and 5 were collapsed into ‘late disease stage’. We then tested the difference between healthy samples versus early disease stage samples (152 samples from N = 37 individuals), healthy samples versus late disease stage samples (107 samples taken from N = 33 individuals), and early disease stage samples versus late disease stage samples (93 samples taken from N = 29 individuals) while controlling for the repeated measures of each individual, with the following model: ~individual + disease_stage_number. For all of these models, the total number of differentially abundant OTUs according to *P*_adj_ < 0.1 were compared to the null distribution of significant OTUs resulting from 1000 permutations where the health status associated with the sample was randomly reassigned. The results of the permutations are presented in Supplementary Table 9. The number of samples included in all statistical tests are presented in Supplementary Table 10. Phyloseq and DESeq2 analyses were performed in R version 3.2.2^86^.

### Functional profiling

Phylogenetic Investigation of Communities by Reconstruction of Unobserved States (PICRUSt)^27^ was used to predict the functional content of the microbial communities. PICRUSt uses extended ancestral-state reconstruction of unknown microbes to microbes with full genome sequences to predict which gene families are present^27^. Of the 1,064 OTUs in our OTU table, 258 were picked with Closed Reference OTU picking and could be used in the PICRUSt analysis (24.2%). Count data for this subset of OTUs was normalized based on 16 s copy number using PICRUSt’s ‘normalize_by_copy_number.py’, functions were predicted using ‘predict_metagenomes.py’, and KEGG Orthology groups (KOs) were collapsed to level 3 with ‘categorize_by_function.py’. To quantify the availability of nearby genome representatives for each microbiome sample (and thus quantify the strength of the predicted functions), PICRUSt calculated a Nearest Sequenced Taxon Index (NSTI). We found the NSTI of our samples to be within the acceptable range for metagenome predictions (mean = 0.08+/−0.04). Differential expression analysis was performed on this biom table using Phyloseq and DESeq2 as explained above.

## Electronic supplementary material

Supplementary Figure S1

Supplementary Tables S1–S10

## Data Availability

The data generated in this study is publicly available through the NCBI Short Read Archive (BioProject ID PRJNA407315). All of the material needed to replicate the analyses in this article has been made publicly available at https://github.com/mlloyd23/SSWD_16S_analysis.
