# Supplementary material for: Microbiome shifts with onset and progression of Sea Star Wasting Disease revealed through time course sampling
Source: Sci Rep. 2018 Nov 7;8:16476. doi: 10.1038/s41598-018-34697-w (PMC6220307; doi:10.1038/s41598-018-34697-w)
Supplement: Supplementary file 1 — Supplementary Figure S1 [file 41598_2018_34697_MOESM1_ESM.pdf]

Supplementary information

Figure S1

Title: Microbiome shifts with onset and progression of Sea Star Wasting Disease revealed through time course sampling

Authors: Melanie M. Lloyd,1* Melissa H. Pespeni,1*

Email addresses: mlloyd@uvm.edu, mpespeni@uvm.edu

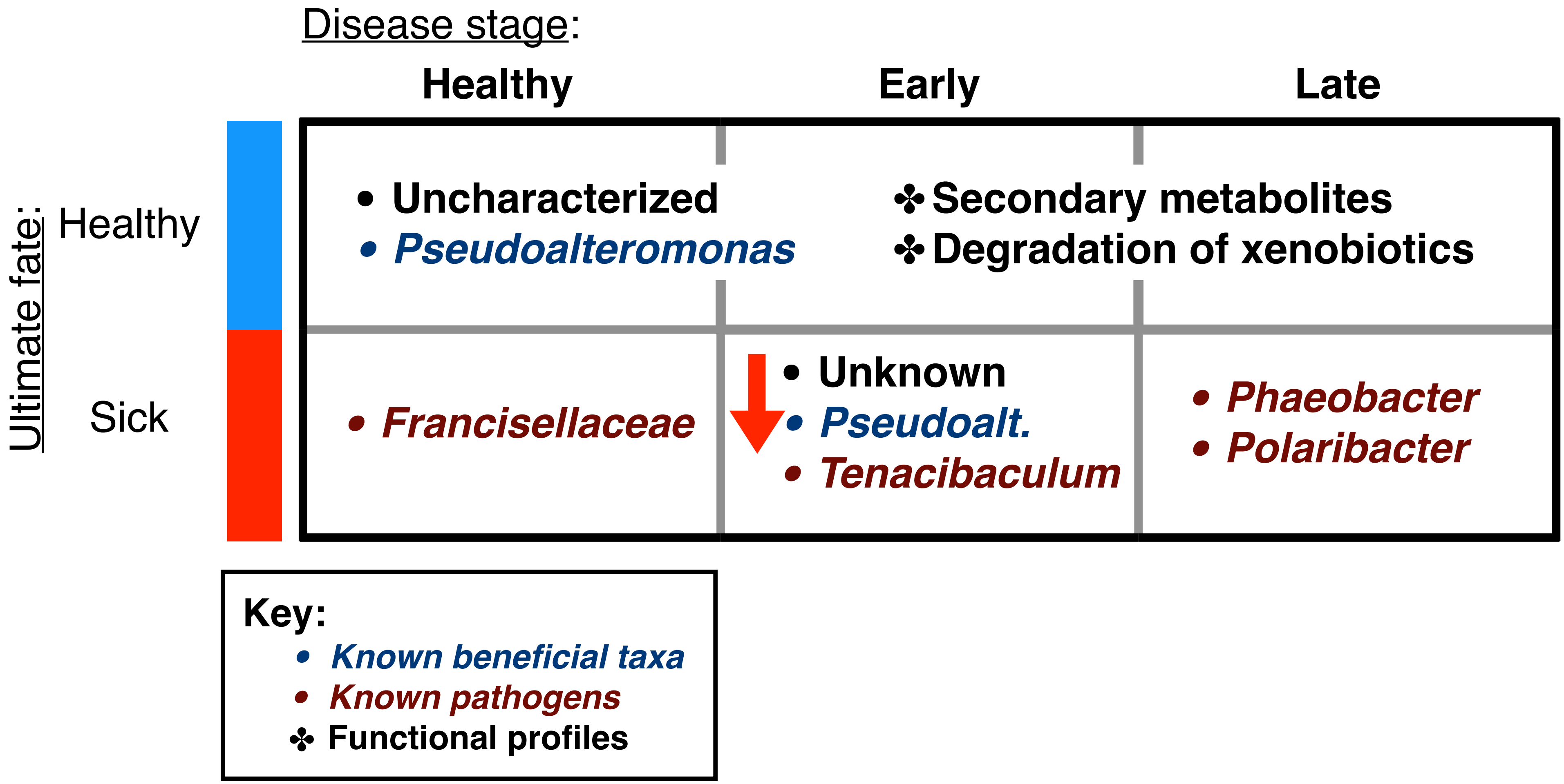

Supplementary Figure S1. Summary schematic of microbiome composition in health and changes in composition through Sea Star Wasting Disease onset and progression. Red arrow indicates decreases in abundance; all other categories represent increases in abundance.
